# Postoperative antibiotic strategies in acute complicated appendicitis: a systematic review

**DOI:** 10.1007/s13304-025-02327-6

**Published:** 2025-07-25

**Authors:** Hussayn Shinwari, Béatrice Marianne Ewalds-Kvist, Michael El Boghdady

**Affiliations:** 1 St George’s University Hospitals NHS Foundation Trust, London, UK; 2 University of Turku, Turku, Finland; 3 Stockholm University, Stockholm, Sweden

**Keywords:** Complicated appendicitis, Postoperative antibiotics, Appendectomy, Appendicectomy, Surgical infections, Acute appendicitis

## Abstract

Acute appendicitis is a common surgical emergency, with complicated cases carrying an increased risk of infections and morbidity. Whilst preoperative antibiotics help reduce infections, the optimal postoperative regimen remains undefined. Variability exists in antibiotic choice, route and duration. This review aimed to examine recent evidence on postoperative antibiotic stewardship for complicated appendicitis to guide optimal treatment strategies. A systematic review was conducted in accordance with PRISMA guidelines and registered in the PROSPERO registry. A search on PubMed and Cochrane library databases identified studies on postoperative antibiotic use in appendicectomy. Two independent reviewers screened studies, including RCTs, cohort studies and observational studies. Data extraction covered study characteristics, interventions and outcomes. Risk of bias was assessed using RoB 2 and ROBINS-I, with GRADE used to evaluate evidence certainty. This review included 11 studies with 8361 participants. Shorter intravenous antibiotic courses (2–6 days) were found to be non-inferior to longer regimens in preventing infections and reducing hospital stays. Risk factors for prolonged antibiotic use included disease severity and surgical complexity. In selected patients, oral antibiotics were shown to be equally effective. Shorter intravenous antibiotic courses and early transition to oral antibiotics effectively managed complicated appendicitis, reducing hospital stays and healthcare costs without increasing complications. Individualised treatment decisions based on patient risk factors and intraoperative findings are essential. Tailoring antibiotic regimens to individual patient characteristics remains crucial. These findings support antibiotic stewardship efforts and highlight the need for further research, particularly in high-risk populations

## Introduction

Acute appendicitis is the most common abdominal surgical emergency worldwide, with an incidence ranging from 96.5 to 100 cases per 100,000 adults annually [1]. Appendicectomy is widely regarded as the gold standard for treating acute appendicitis [2]. Complications following appendicectomy, such as intra-abdominal abscesses, are reported in 4%–28% of cases, whilst surgical-site infections (SSI) affect up to 11% of patients [3, 4]. Less common, including postoperative ileus and bowel obstruction, can result in unplanned readmissions in approximately 10% of cases [5]. Complicated appendicitis (CA) including perforated appendicitis, gangrenous or appendicitis with abscess formation presents a greater challenge in management, increased patients’ morbidity and infectious complications [2, 6].

Preoperative administration of broad-spectrum antibiotics has been shown to be effective in decreasing the risk of wound infections and abscess formation [7]. The preoperative antibiotics are universally administered to reduce the risk of SSIs and intra-abdominal infections [8, 9]. In healthy young adult patients, opportunities remain for improvement in the choice of postoperative antibiotic stewardship to timely discontinue prophylactic antibiotics and implement enhanced recovery and ambulatory treatment pathways for uncomplicated appendicitis [10]. However, there remains a lack of consensus regarding the optimal postoperative antibiotic regimen for complicated appendicitis. There is considerable variability in the route of administration, different agents, dose and duration of antibiotics [11].

Given the global variation in recommended durations of antibiotic therapy, the optimal postoperative management of CA remains undefined. Therefore, this systematic review aimed to study newer literature regarding the postoperative antibiotics’ stewardship for complicated appendicitis. Consequently, we posed the following research questions:

1. Is there evidence that a longer course of IV antibiotics provides benefits compared to a shorter course after complicated appendicitis in selected patients?
2. Does a longer course of IV antibiotics reduce the risk of postoperative surgical infections (PSI) more than that of a shorter course?
3. Is a longer postoperative antibiotic course in selected patients associated with additional risk factors?
4. Is there a difference in outcomes between oral and IV antibiotics in patients with complicated appendicitis?

## Methodology

The review adhered to the PRISMA (Preferred Reporting Items for Systematic Reviews and Meta-Analyses) guidelines and was registered in PROSPERO (CRD42024559392) [12].

### Search strategy and study selection

A comprehensive literature search was conducted using PubMed and The Cochrane library for studies published between August 31, 2019, and August 31, 2024. The search strategy employed a combination of MeSH terms and keywords, including “appendicitis,” OR “appendicectomy,” OR “appendectomy,” OR “append*,” AND “antibiotic,” OR “antibiotics,” OR “antibacterial,” OR “antimicrobial.”

**Fig. 1:**
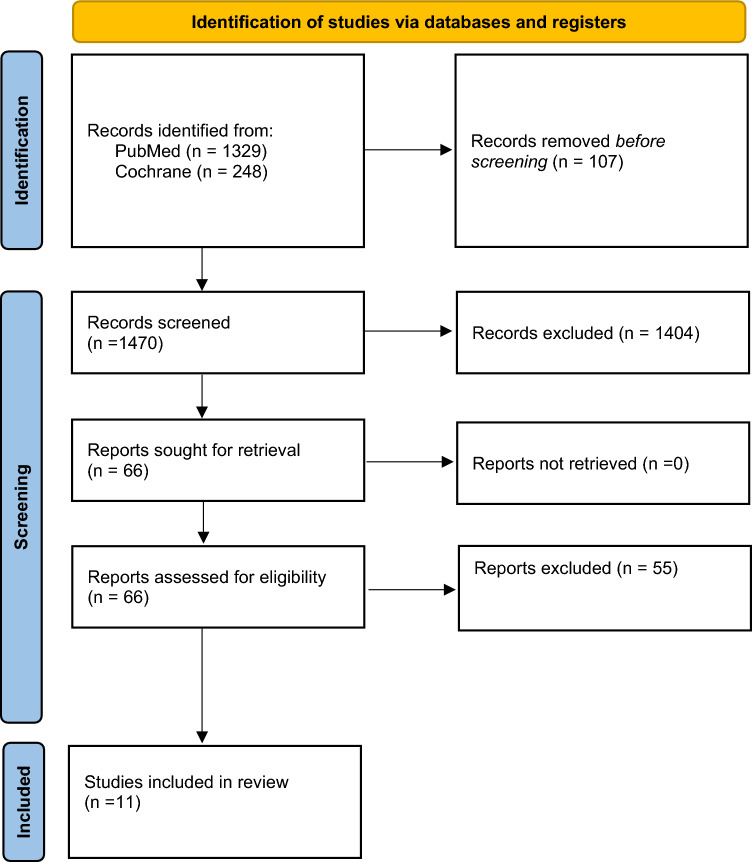
PRISMA flow diagram for the systematic review

The study selection process followed a structured approach, with titles, abstracts and full-text articles screened sequentially by two independent reviewers. Any disagreements during the selection process were resolved through discussion and consensus. To ensure relevance, the inclusion criteria targeted studies involving adult patients undergoing appendicectomy, either laparoscopic or open, that studied the use of antibiotics with no antibiotics following surgery. Studies were required to report at least one of the primary outcomes: morbidity, complications or mortality. Eligible designs included randomised controlled trials (RCTs), cohort studies and observational studies. Paediatric studies and those not directly addressing antibiotic use post-appendicectomy were excluded. Similarly, letters, commentaries, case reports, editorials, technical reports, conference abstracts, reviews and articles in non-English language were not considered for inclusion (Fig. 1).

### Data extraction

Data extraction was performed using a pre-defined standardised form to ensure consistency and accuracy. Two independent reviewers extracted key information, including study characteristics (author, publication year, journal, country), study design, sample size, demographic details, type and route of antimicrobial therapy, comparator group details, and primary outcomes (morbidity, complications, mortality).

### Data analysis

Extracted data were organised and analysed using Microsoft Excel 2024. Studies were categorised based on their characteristics, the nature of the intervention and reported outcomes. Heterogeneity amongst studies was assessed qualitatively through a tabular comparison of key study attributes. Results were presented through cohort diagrams, summary tables and detailed narrative descriptions.

### Quality strategy and study selection

The certainty of evidence for each outcome was assessed using the GRADE (Grading of Recommendations, Assessment, Development, and Evaluation) approach [13]. Evidence from randomised controlled trials (RCTs) and non-randomised studies (NRS) was evaluated separately, following the GRADE framework.

### Risk of bias assessment

RCTs were assessed using the RoB 2 tool, classifying studies as having a low, some concerns or high risk of bias across multiple domains.

NRS were assessed using the ROBINS-I tool, categorising studies into low, moderate, serious or critical risk of bias across seven domains. Studies classified as having a critical risk of bias using ROBINS-I were excluded from evidence synthesis, in line with GRADE guidelines.

### Classification of evidence by GRADE

For each outcome, the certainty of evidence was determined using the following GRADE framework:

- High certainty: Further research is unlikely to change confidence in the effect estimate. This level was assigned when evidence was based on at least two high-quality RCTs or a systematic review/meta-analysis of high evidential value.
- Moderate certainty: Further research may impact confidence in the estimate. This level included one high-quality RCT plus additional moderate-quality studies or multiple NRS with low risk of bias.
- Low certainty: Further research is likely to have a significant impact on the effect estimate. This level included studies with moderate or serious risk of bias, including NRS.
- Very low certainty: Evidence was deemed unreliable due to high risk of bias, serious inconsistency, or imprecision.

## Results

All together 8361 participants were involved in the present systematic review comprising 11 studies about postoperative care of acute complicated appendicitis (CA) [14–24].

**Fig. 2:**
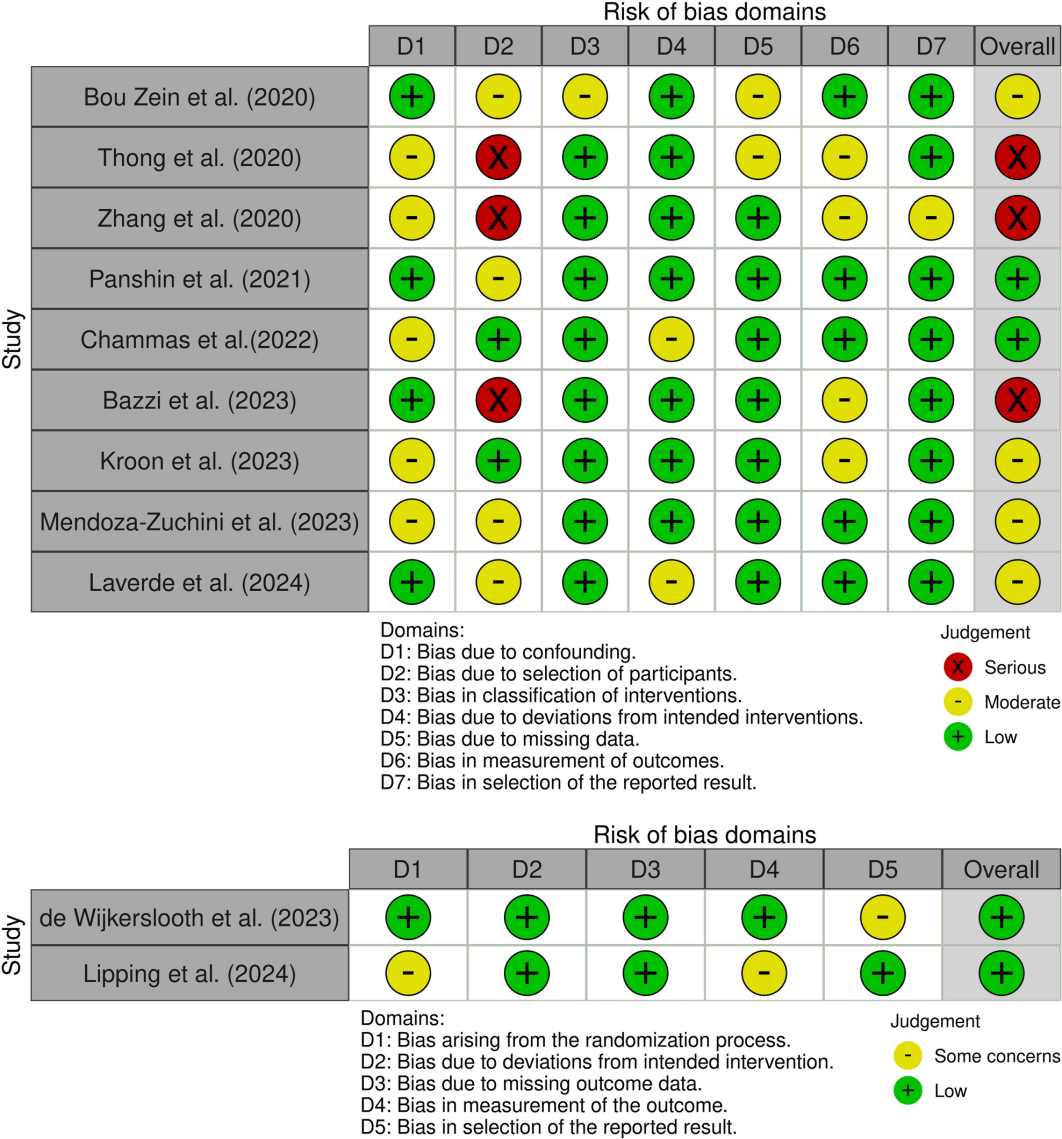
Risk of bias across the included studies

The authors’, year, and type of publication, as well as the journals are presented in Table 1. Further, the definition of complicated appendicitis in each study, guidelines/protocols, aim, endpoints, and the main findings of each of the citations were simplified. Types of antibiotics, duration, route, postoperative complications, length of hospital stay, follow-up and readmission were studied and compared between included studies, as shown in Table 2. Risk of bias assessment was analysed amongst the included studies (Fig. 2).

**Table 1:** Summary of included studies on postoperative antibiotics in complicated appendicitis

Author (Year)	Study type	Study aim	Definition of complicated appendicitis (CA)	Guidelines referenced	Primary endpoints	Secondary endpoints	Main findings	GRADE certainty
Bou Zein et al. (2020)	Prospective multi-centre	Compare ≤24-hour vs ≥96-hour IV antibiotics for complicated appendicitis	Gangrenous or perforated appendicitis	American Association for the Surgery of Trauma (AAST)	Surgical-site infection (SSI), length of hospital stay (LOS)	Postoperative surgical infections (PSIs)	No difference in SSI rate; ≤24-hour group had shorter hospital stay	Moderate
Thong et al. (2020)	Multi-centre observational	Assess compliance to ABx guidelines and complications post-appendicectomy	Perforation, empyema, periappendiceal abscess, or faeculent peritonitis	Australian Therapeutic Guidelines (TGA v15)	Postoperative surgical infections (SSI and intra-abdominal abscess)	Not clearly reported	Inconsistent guideline adherence; varied SSI and IAA rates	Low
Zhang et al. (2020)	Retrospective cohort	Identify factors influencing hospital stay duration post-appendicectomy	Ambiguously defined; included acute appendicitis in adults >15 to <80 years without negative findings	SAGES, UpToDate	Length of hospital stay (LOS), postoperative surgical infections (PSIs)	Risk factors for LOS (age, peritonitis, surgical delay)	Older age, peritonitis, delayed surgery linked to longer LOS	Low
Panshin et al. (2021)	Retrospective cohort	Assess optimal antibiotic duration to reduce complications in CA	Complicated appendicitis was defined as operative Grades II–V according to the AAST grading system.	SIS, IDSA (2010)	PSIs, LOS, deep space infections (DSIs)	Impact of antibiotic duration by operative grade	3–6 days of antibiotics recommended for optimal outcomes	Moderate
Chammas et al. (2022)	Post hoc comparative	Evaluate outcomes of restricted vs. liberal ABx post-appendicectomy	Perforated or gangrenous appendicitis	Eastern Association for the Surgery of Trauma (EAST)	PSIs, LOS	Comparison of restricted vs. liberal antibiotic use	Restricted ABx use associated with better outcomes	Moderate
Bazzi et al. (2023)	Retrospective single-centre	Compare characteristics and outcomes in simple vs. complicated appendicitis	Not clearly defined; related to severity in economic crisis	World Society of Emergency Surgery (WSES) Jerusalem (2020)	Rate and characteristics of complicated appendicitis	Surgery timing, complications, LOS, ABx use	Antibiotic use and LOS related to disease severity	Low
de Wijkersloot et al. (2023)	Pragmatic multi-centre RCT	Compare 2-day vs 5-day IV ABx regimens in CA	Necrosis, perforation, or abscess	Dutch Surgical Association	Composite: infectious complications and mortality within 90 days	SSI, LOS, adjusted risk difference	2-day IV ABx non-inferior to 5-day for infection prevention	High
Kroon et al. (2023)	Retrospective cohort	Evaluate short-course IV ABx for CA	Gangrenous or perforated appendix at surgery	SIS, IDSA, Australia TGA	PSIs, LOS, 30-day unplanned readmission	Safety of short-course IV antibiotics	Short IV course safe; no increase in readmission	Moderate
Mendoza-Zuchini et al. (2023)	Prospective cohort	Assess ABx approach based on clinical response	Perforation, phlegmon, abscess, or peritonitis	SIS, IDSA, WSES	PSI rate, mortality; comparison of IV vs oral antibiotic approach	SSI, rehospitalisation, treatment cost	No difference in SSI, rehospitalisation or costs	Moderate
Laverde et al. (2024)	Retrospective cohort	Determine ideal ABx duration to reduce IAA and wound infections	Transmural inflammation with necrosis, perforation, and/or intra-abdominal abscess	SAGES, UpToDate	PSIs, LOS, intra-abdominal abscess (IAA), wound infections	Effect of shorter antibiotic duration	Short ABx duration did not increase IAA or wound infections	Moderate
Lipping et al. (2024)	Pilot RCT (non-inferiority)	Compare post-operative oral vs. IV ABx in CA	Gangrenous, perforated, or with periappendicular abscess	SAGES, UpToDate	30-day complications per Comprehensive Complication Index	SSI, re-consultation, LOS	24-hour oral ABx non-inferior to 24 hour IV ABx post-operatively	High

*ABx* antibiotics, *AAST* American Association for the Surgery of Trauma, *CAA* acute complicated appendicitis, *DSI* deep space infection, *EAST* Eastern Association for the Surgery of Trauma, *GRADE* Grading of Recommendations Assessment, Development and Evaluation, *IAA* intra-abdominal abscess, *IDSA* Infectious Diseases Society of America, *IV* intravenous, *LOS* length of stay, *PSI* postoperative surgical infection, *RCT* randomised controlled trial, *SAGES* Society of American Gastrointestinal and Endoscopic Surgeons, *SIS* Surgical Infection Society, *SSI* surgical-site infection, *TGA* Therapeutic Guidelines Australia, *UpToDate* A Clinical Decision Support Resource, *WSES* World Society of Emergency Surgery

**Table 2:** Antibiotic use, route, LOS, follow-up and readmission in included studies

Author (year)	Type of antibiotics	Route	Length of hospital stay (LOS)	Follow-up	Readmission
Bou Zein et al. (2020)	Zosyn, Augmentin, metronidazole, fluoroquinolones, 3rd-gen cephalosporins, others	IV and/or oral depending on severity and agent	≤24 hours: Median 1 day (IQR 1–2), ≥96 hours: Median 4 days (IQR 2–6), p ≤ 0.0001	30-day follow-up	Readmission: 9% (no significant difference between ≤24 and ≥96 -hour)
Thong et al. (2020)	Gentamicin, amoxicillin, metronidazole, oral Augmentin	IV followed by oral in ~30% patients	N/A	30-day follow-up	N/A
Zhang et al. (2020)	Ceftriaxone + metronidazole, ertapenem	IV	LOS ≤3 days (348 pts), LOS >3 days (288 pts)	N/A	N/A
Panshin et al. (2021)	Piperacillin/tazobactam, ertapenem, Cefoxitin	IV	<3 days: Median 1.0 (IQR 1.22), 3–4 days: Median 3.2 (IQR ~1.62), 5–6 days: Median 3.1 (IQR ~2.7), 6 days: Median 4.7 (IQR 6.75)	N/A	<3d: 11.3%, 3–4d: 15.7%, 5–6d: 3.8%, >6d: 8%
Chammas et al. (2022)	Not specified	N/A	Restricted ABx use favoured for efficiency	1-year follow-up	N/A
Bazzi et al. (2023)	N/A	IV for all; PO in simple cases only	Simple: Median 2.69 days (IQR= 1,24), CA: Median 3.88 days (IQR= 2.5) , *p* < 0.001	N/A	N/A
de Wijkerslooth et al. (2023)	Cefuroxime/ceftriaxone + metronidazole; some oral follow-up	IV ± oral (protocol violations)	2-day group: Median 3 days (2–4), 5-day group: Median 5 days (5–6), *p* < 0.001	90-day follow-up (phone)	2d: 12%, 5d: 6%, OR 2.135 (95% CI 1.342–3.396)
Kroon et al. (2023)	Amoxicillin/clavulanic acid	IV → Oral (based on response)	Short:Median 2.1 days (IQR 2), long: Median 6.5 days (IQR 14)	30-day follow-up	Short: 7%, Long: 6%, *p* = 0.99
Mendoza-Zuchini et al. (2023)	Ampicillin–sulbactam, clindamycin + aminoglycosides	IV → Oral (based on response)	Same-day: 23%, 1 day: 25%, 2 days: 14.5%, 3 days: 27%, >3 days: 10.4%	N/A	2 readmissions; no significant difference (*p* = 0.44)
Laverde et al. (2024)	Cefotaxime + metronidazole or piperacillin/tazobactam	IV; 15% oral on discharge	Group 1: Median 4 days (IQR: 2)., Group 2: Median 6 days (IQR: 4), *p* < 0.001	90-day follow-up	Group 1: 4%, Group 2: 6%
Lipping et al. (2024)	Amoxicillin/clavulanic acid	IV or oral (24 hours post-operatively)	No significant LOS difference.IV: Median 1.2 days (IQR 0.6) , oral: Median 1.3 days (IQR 0.8)	30-day follow-up	IV: 10%, oral: 9%

*IV* intravenous, *PO* per Os (by mouth/oral), *LOS* length of hospital stay, *IQR* interquartile range, *CA* complicated appendicitis, *ABx* antibiotics, *OR* odds ratio, *CI* confidence interval, *N/A* not available

### Application of GRADE to included studies

The overall certainty of evidence was graded for each outcome based on study design, risk of bias, inconsistency, indirectness, imprecision and potential publication bias, following GRADE recommendations. The distribution of studies contributing to the evidence certainty for different outcomes was as follows:

High certainty of evidence: de Wijkerslooth et al. [20]; Lipping E et al. [23].

Moderate certainty of evidence: Studies with moderate risk of bias but still providing valuable data included Laverde et al., Panshin et al., Kroon HM et al., Chammas et al., Mendoza-Zuchini et al., and Bou Zein et al.

### Research questions and answers

1. Is there evidence that a longer course of IV antibiotics provides benefits compared to a shorter course after complicated appendicitis in selected patients?

De Wijkerslooth et al. demonstrated that 2 days of postoperative intravenous antibiotics for complicated appendicitis is non-inferior to 5 days in terms of infectious complications and mortality within 90 days [20]. In agreement, Kroon et al. [19] and Panshin et al. [17] confirmed that given the lower incidence of postoperative complications between 3–6 days and with no added benefit > 6 days, a limitation of antibiotic treatment to 3–6 days for all CA cases can be recommended.

Furthermore, Lipping et al. administered to 51 patients with CA, a 24-hour intravenous antibiotics and compared them to 53 patients with a 24-hour oral treatment group with similar disease severity scores for acute complicated appendicitis [23]. There were no differences between the groups with regard to 30-day postoperative complications. In addition, Median Comprehensive Complication Index was similar between the two groups.

Bou Zein Eddine et al. [16] reported no significant difference in surgical-site infection (SSI) rates comparing ≤24-hour and ≥96-hour antibiotic courses. However, longer courses of antibiotics were linked to more complications, with a 15% intra-abdominal abscesses rate in the extended antibiotic group compared to 7% in the shorter group (*p* ≤ 0.0001), along with longer hospital stays. An analysis of longer vs. shorter IV antibiotic courses can be found in Table 3.

**Table 3:** Longer vs shorter IV antibiotic courses in complicated appendicitis

Study	Short duration	Long duration	Complication rate	LOS (short vs long)	Readmission rate	Conclusion
Bou Zein et al. (2020)	≤24-hour IV	≥96-hour IV	Higher in long course	1 day vs. 4 days	8% vs. 9% (not significant)	Shorter duration linked to fewer abscesses and shorter LOS.
Panshin et al. (2021)	<3 days IV	≥6 days IV	Lower in 5–6 day group	1 day vs. 4.7 days	11.3% vs. 8%	3–6 day course may be optimal; longer duration showed no added benefit.
Kroon et al. (2023)	~2.1 days IV	~6.5 days IV	Similar	2.1 days vs. 6.5 days	7% vs. 6%	Short course was safe and reduced LOS without increasing complications.
de Wijkerslooth et al. (2023)	2 days IV	5 days IV	Similar	3 days vs. 5 days	12% vs. 6% (↑ in 2-day group)	2-day IV was non-inferior to 5-day; slightly higher readmissions in 2-day group.

*LOS* = length of stay, *IV* = intravenous,

2. Does a longer course of IV antibiotics reduce the risk of postoperative surgical infections (PSI) and length of hospital stay (LOS) more  than a shorter course in selected patients?

De Wijkerslooth et al. found that switching from 5 days to 2 days courses of postoperative antibiotics saves direct healthcare costs and indirect societal costs mainly related to reduced hospital stays, whereas there was no significant difference in costs related to productivity losses (i.e. sick leave) [20]. Eventually, restriction of antibiotics results in reduced length of hospital stay without a compromise in infectious complications or re-interventions.

Kroon et al. reported that short course of IV antibiotics following CA is safe and cut the LOS in patients with tailored antibiotic therapy involving surgeon’s supervision of the patient’s responses and clinical progress as well as his/her adjustment of the duration of antibiotic treatment to optimise outcomes and minimise risks [19]. In these selected patients, a short course of IV antibiotics does not raise the risk of post-operative infections or unplanned readmission within 30 days. In accordance, Bou Zein et al. revealed that a shorter antibiotic duration was linked to a shorter hospital stay in selected patients [16].

3. Is a longer postoperative antibiotic course in selected patients associated with additional  risk factors?

De Wijkerslooth et al. randomised the included patients and compared those who got 2 days antibiotics with those patients who got 5 days antibiotics post-surgery [20]. Although deviations in antibiotic administration were allowed, the study found that 2 days of antibiotics was non-inferior to 5 days, based on a non-inferiority margin of 7.5%. However, these findings are applicable primarily to well-resourced healthcare settings, and may not generalise to low-resource environments.

Kroon et al. confirmed that intraoperative findings of the appendix constituted a prognostic factor for post-surgery infections and ASA scores, as well as surgical approach represented the prognostic predictors of 30-day unplanned readmission [19]. Bou Zein Eddine et al. [16] found that patients with CA who received  ≥96 hours of antibiotics had significantly higher rates of intra-abdominal abscesses. Panshin et al. [17] reported that patients with complicated appendicitis required antibiotics for 8–10 days, and that higher surgical grades correlated with longer antibiotic durations[17]. In summary, extended postoperative antibiotic courses are typically prescribed for patients presenting with additional risk factors, such as greater disease severity, intraoperative complications, and higher ASA scores, all of which warrant tailored management strategies.

4. Is there a difference in outcomes between oral and IV antibiotics in patients with complicated appendicitis?

Lipping, et al. found that oral antibiotic administration resulted in non-inferior outcomes compared with the 24-hour IV antibiotics administration after laparoscopic appendicectomy in complicated cases [23].

Kroon et al. found that postoperative IV antibiotics can safely be switched to oral antibiotics (amoxicillin/clavulanic acid 875/125 mg) when, after 48 hours, patients are responding well to therapy, which is determined as being afebrile and having a decreasing white cell count. Patients can then be discharged home [19].

Laverde et al.’s cohort of 394 patients with CA were treated post-surgery with the standard postoperative antibiotic regimen consisting of either cefotaxime (2 g, t.i.d.) and metronidazole (500 mg, t.i.d.) or piperacillin/tazobactam (4.5 g, t.i.d.) [24]. Oral antibiotic therapy was continued after hospital discharge in 61 patients (15%). The duration of antibiotic therapy was determined collaboratively by the surgeon and the attending physicians on the ward, considering the results of the intraoperative swab and the patient’s clinical condition. Oral antibiotic therapy was considered to be equivalent to IV antibiotics in terms of clinical outcomes in postoperative care.

## Discussion

Antibiotic prescription plays a crucial role in the management of acute complicated appendicitis, particularly in preventing postoperative complications. Preoperative antibiotic administration is strongly recommended once the diagnosis of acute appendicitis is confirmed, as it helps reduce the risk of infection during and after surgery. However, there has been an ongoing debate regarding antibiotic stewardship in the postoperative setting, particularly concerning the optimal route and duration of antibiotic therapy. Whilst prolonged courses of intravenous antibiotics were historically favoured, newer evidence suggests that shorter durations, or even an early switch to oral antibiotics, may be equally effective in selected patients with complicated appendicitis. This shift in approach aims to reduce unnecessary antibiotic prescription, reduce healthcare costs and shorten hospital stays whilst ensuring patient safety [25].

The optimal route of antibiotic prescription in acute complicated appendicitis remains a subject of debate, balancing efficacy, patient outcomes and antibiotic stewardship. Traditionally, intravenous (IV) antibiotics have been the standard approach postoperatively, given their reliable bioavailability and effectiveness in severe infections. However, emerging evidence suggests that early transition to oral antibiotics, or even exclusive oral regimens in selected patients, may be equally effective whilst reducing hospital stays, healthcare costs and the risks associated with prolonged IV therapy. Studies have demonstrated non-inferior outcomes with oral antibiotics compared to IV administration, particularly when patients are clinically stable, afebrile, and showing signs of recovery within 24–48 hour post-surgery. Despite these findings, concerns persist about ensuring adequate absorption and compliance with oral therapy, particularly in patients with severe intra-abdominal infections. As a result, clinical decisions regarding antibiotic route should be individualised, taking into account patient-specific factors, intraoperative findings and response to initial treatment [26, 27].

The optimal duration of antibiotic therapy in complicated acute appendicitis remains a topic of enduring discussion, with current evidence favouring shorter courses in selected patients. Traditionally, extended IV antibiotic regimens were used postoperatively to prevent infectious complications. However, studies [17, 19, 20] have demonstrated that limiting IV antibiotics to 3–6 days, or even as short as to 2 days, does not increase the risk of postoperative infections or mortality. Yet, Lipping et al. showed that a 24-hour IV antibiotic course followed by oral antibiotics produced comparable outcomes equated to prolonged IV therapy [23]. Shorter antibiotic regimens have also been linked to reduced healthcare costs and shorter hospital stays without compromising patient safety [15, 16]. However, patient selection is crucial, as intraoperative findings, ASA scores, and surgical approach influence the need for extended antibiotic therapy. Therefore, whilst evidence supports a shift toward shorter antibiotic courses, the duration should be tailored based on individual patient risk factors and clinical response [25].

The length of hospital stay (LOS) after acute complicated appendicitis is closely linked to postoperative complications and the duration of antibiotic therapy. It has been shown that shorter antibiotic courses can safely reduce LOS without increasing the risk of complications [20]. Reducing postoperative IV antibiotics from 5 days to 2 days led to shorter hospital stays and lower healthcare costs without compromising infection rates or the need for reintervention. Similarly, Kroon et al. and Bou Zein Eddine et al. found that limiting antibiotic duration contributed to a reduced LOS whilst maintaining patient safety [16, 19]. Zhang et al. found that specific antibiotic regimens, such as cephalosporins plus metronidazole, were associated with shorter hospital stays when aligned with national healthcare policies [15]. However, postoperative complications remain a significant factor influencing LOS, as patients with higher ASA scores, severe intraoperative findings or inadequate initial treatment may require extended hospitalisation. Overall, whilst a shorter LOS is desirable, it should not come at the expense of patient safety, making individualised treatment decisions essential [26].

One limitation of this study is the inclusion of some paediatric patients amongst the participants in a few citations, which may impact the generalisability of the findings to an exclusively adult population. Paediatric patients often necessitate different antibiotic regimens and treatment approaches compared to adults due to variations in physiology, immune response and risk factors for complications. Furthermore, differences in antibiotic selection, dosing and duration between paediatric and adult populations could introduce heterogeneity in the results. Future studies should consider stratifying outcomes by age group to provide more precise recommendations tailored to distinct patient populations.

The clinical implications of this study emphasise the potential for optimising antibiotic stewardship in the management of acute complicated appendicitis. The findings support the accumulating evidence that shorter courses of intravenous (IV) antibiotics or an early transition to oral antibiotics can be equally effective in selected patients, thereby reducing hospital stays, healthcare costs and the risks associated with prolonged IV therapy. This underscores the importance of individualised treatment, where the duration and route of antibiotic administration should be guided by patient-specific factors such as intraoperative findings, comorbidities and clinical response. Moreover, the study highlights the necessity for future randomised trials with careful patient selection, particularly in vulnerable populations such as paediatric and elderly patients, who may require different antibiotic regimens. Implementing these findings in clinical practice could enhance patient outcomes, minimise unnecessary antibiotic use and contribute to global efforts in antimicrobial resistance prevention.

## Conclusion

The findings of this study suggested that a longer course of IV antibiotics offers no significant benefits over a shorter course in selected patients with acute complicated appendicitis. Additionally, oral antibiotic therapy has demonstrated non-inferior outcome compared to IV therapy when administered appropriately, further supporting the shift towards more conservative antibiotic strategy. Postoperative antibiotic use should be individualised based on intra-operative findings, patient risk factors, and clinical response.

## Data Availability

All data analysed in this systematic review are publicly available from the cited original studies.

## References

[1] Moris D, Paulson EK, Pappas TN (2021) Diagnosis and Management of Acute Appendicitis in Adults: A Review. JAMA. 326:1 10.1001/jama.2021.20502 34905026

[2] Bhangu A, Søreide K, Di Saverio S, Assarsson JH, Drake FT (2015) Acute appendicitis: Modern understanding of pathogenesis, diagnosis, and management. Lancet. 386:1 26460662 10.1016/S0140-6736(15)00275-5

[3] Levin DE, Pegoli W (2015) Abscess After Appendicectomy: Predisposing Factors. Adv Surg. 49:1 26299504 10.1016/j.yasu.2015.03.010

[4] Zamaray B, de Boer MFJ, Popal Z, Rijbroek A, Bloemers FW, Oosterling SJ (2023) AbcApp: incidence of intra-abdominal ABsCesses following laparoscopic vs. open Appendicectomy in complicated appendicitis. Surg Endosc. 37(3):1 36203108 10.1007/s00464-022-09670-4 PMC10017785

[5] Moghadamyeghaneh Z, Hwang G, Hanna MH, Carmichael JC, Mills S, Pigazzi A et al (2016) Unplanned readmission after appendicectomy. Am J Surg. 1:1 10.1016/j.amjsurg.2015.08.018 26602535

[6] Mariage M, Sabbagh C, Grelpois G, Prevot F, Darmon I, Regimbeau JM (2019) Surgeon’s Definition of Complicated Appendicitis: A Prospective Video Survey Study. Euroasian J Hepatogastroenterol. 9(1):1 31988858 10.5005/jp-journals-10018-1286 PMC6969325

[7] Andersen BR, Kallehave FL, Andersen HK (2005) Antibiotics versus placebo for prevention of postoperative infection after appendicectomy. Cochrane Database Syst Rev. 2009(1):1 10.1002/14651858.CD001439.pub2 PMC8407323 16034862

[8] Wagner JM, McKinney WP, Carpenter JL (1996) Does this patient have appendicitis? JAMA. 276:1 8918857

[9] Benabbas R, Hanna M, Shah J, Sinert R (2017) Diagnostic Accuracy of History, Physical Examination, Laboratory Tests, and Point-of-care Ultrasound for Pediatric Acute Appendicitis in the Emergency Department: A Systematic Review and Meta-analysis. Acad Emerg Med. 24(5):1 10.1111/acem.13181 28214369

[10] Di Saverio S, Birindelli A, Kelly MD, Catena F, Weber DG, Sartelli M et al (2016) WSES Jerusalem guidelines for diagnosis and treatment of acute appendicitis. World J Emerg Surg 11:1 27437029 10.1186/s13017-016-0090-5 PMC4949879

[11] El Boghdady M (2024) Pre- and Post-operative Antibiotics for Acute Appendicitis: Review of the Recent Recommendations. Indian J Surg. 11:1

[12] Page MJ, McKenzie JE, Bossuyt PM, Boutron I, Hoffmann TC, Mulrow CD, Moher D (2021) The PRISMA 2020 statement: an updated guideline for reporting systematic reviews. BMJ. 372:1 10.1136/bmj.n71 PMC8005924 33782057

[13] Granholm A, Alhazzani W, Møller MH (2019) Use of the GRADE approach in systematic reviews and guidelines. Br J Anaesth 123(5):554–559 31558313 10.1016/j.bja.2019.08.015

[14] QUEST Collaboration, Thong DW, Kim J, Dobson B, Cheung H, Arthur T (2020) Variation in anti-microbial prescription and complications post emergency appendicectomy in Australia: do we follow recommended guidelines? ANZ J Surg. 90(3):251 30776854 10.1111/ans.15099

[15] Zhang P, Zhang Q, Zhao H, Li Y (2020) Factors affecting the length of hospital stay after laparoscopic appendicectomy: A single center study. PLoS One. 15(12):e0243575 33296384 10.1371/journal.pone.0243575 PMC7725291

[16] EAST Appendicitis Research Group, Bou Zein ES, Dodgion CM, Qian S, Trevino C, De Moya MA, Yeh DD (2020) Complicated Appendicitis: Are Extended Antibiotics Necessary? A Post Hoc Analysis of the EAST Appendicitis “MUSTANG” Study. J Surg Res. 247:508 31812337 10.1016/j.jss.2019.09.054

[17] Panshin MS, Alnachoukati OK, Schroeppel TJ, Metzler M, McFann K, Dunn JA (2021) Optimal duration of antibiotics following appendicectomy for patients with complicated appendicitis. Am Surg. 87(3):480–5. 10.1177/0003134820947372. (**Epub 2020 Oct 13 PMID: 33047976**) 33047976 10.1177/0003134820947372

[18] EAST Appendicitis Study Group, Chammas M, Pust GD, Hatton G, Pedroza C, Kao L, Rattan R, Namias N, Yeh DD (2022) Outcomes of restricted versus liberal post-operative antibiotic use in patients undergoing appendicectomy: A DOOR/RADAR post hoc analysis of the EAST Appendicitis MUSTANG study. Surg Infect (Larchmt). 23(5):489–494. 10.1089/sur.2021.287. (**PMID: 35647893**) 35647893 10.1089/sur.2021.287

[19] Kroon HM, Kenyon-Smith T, Nair G, Virgin J, Thomas B, Juszczyk K, Hollington P (2023) Safety and efficacy of short-course intravenous antibiotics after complicated appendicitis in selected patients. Acta Chir Belg. 123(1):49–53. 10.1080/00015458.2021.1940726. (**Epub 2021 Jul 29 PMID: 34120572**) 34120572 10.1080/00015458.2021.1940726

[20] APPIC Study Group, de Wijkerslooth EML, Boerma EG, van Rossem CC, van Rosmalen J, Baeten CIM, Beverdam FH et al (2023) 2 days versus 5 days of postoperative antibiotics for complex appendicitis: a pragmatic, open-label, multicentre, non-inferiority randomised trial. Lancet. 401(10374):366–76. 10.1016/S0140-6736(22)02588-0. (**Epub 2023 Jan 17 PMID: 36669519**) 36669519 10.1016/S0140-6736(22)02588-0

[21] Bazzi N, Dbouk S, Rached A, Jaber S, Bazzi H, Jrad M, Bazzi M (2023) An update on acute appendicitis in Lebanon: insights from a single-center retrospective study. Cureus. 15(5):e38792. 10.7759/cureus.38792.PMID:37303416;PMCID:PMC10250019 37303416 10.7759/cureus.38792 PMC10250019

[22] Mendoza-Zuchini A, Arce-Polania LC, Pérez-Rivera CJ (2023) Intravenous antibiotic therapy after laparoscopic appendicectomy in acute complicated appendicitis: the patient clinical response is the key. Cir Cir. 91(4):479–85. 10.24875/CIRU.21000557. (**PMID: 37677930**) 37677930 10.24875/CIRU.21000557

[23] Lipping E, Saar S, Reinsoo A, Bahhir A, Kirsimägi Ü, Lepner U et al (2024) Short postoperative intravenous versus oral antibacterial therapy in complicated acute appendicitis: a pilot noninferiority randomized trial. Ann Surg. 279(2):191–5. 10.1097/SLA.0000000000006103. (**Epub 2023 Sep 25 PMID: 37747168**) 37747168 10.1097/SLA.0000000000006103

[24] Laverde BLB, Maak M, Langheinrich M, Kersting S, Denz A, Krautz C, Weber GF, Grützmann R, Brunner M (2024) Antibiotic treatment after appendicectomy for acute complicated appendicitis to prevent intra-abdominal abscess and wound infections. Langenbecks Arch Surg. 409(1):180 38850459 10.1007/s00423-024-03367-z PMC11162365

[25] Van den Boom AL, de Wijkerslooth EML, Giesen LJX, van Rossem CC, Toorenvliet BR, Wijnhoven BPL (2022) Postoperative antibiotics and time to reach discharge criteria after appendicectomy for complex appendicitis. Dig Surg. 39(4):162–168. 10.1159/000526790 36041400 10.1159/000526790 PMC9909712

[26] Oba T, Yamada T, Matsuda A, Otani M, Matsuda S, Ohta R, Yoshida H, Sato N, Hirata K (2021) Patient backgrounds and short-term outcomes of complicated appendicitis differ from those of uncomplicated appendicitis. Ann Gastroenterol Surg. 6(2):273–81. 10.1002/ags3.12523 35261953 10.1002/ags3.12523 PMC8889856

[27] Cyriac JM, James E (2014) Switch over from intravenous to oral therapy: A concise overview. J Pharmacol Pharmacother. 5(2):83–7. 10.4103/0976-500X.130042 24799810 10.4103/0976-500X.130042 PMC4008927

